# Supplementary figures and images for: A novel kinetic energy harvesting system for lifetime deployments of wildlife trackers
Source: PLoS One. 2023 May 17;18(5):e0285930. doi: 10.1371/journal.pone.0285930 (PMC10191315; doi:10.1371/journal.pone.0285930)

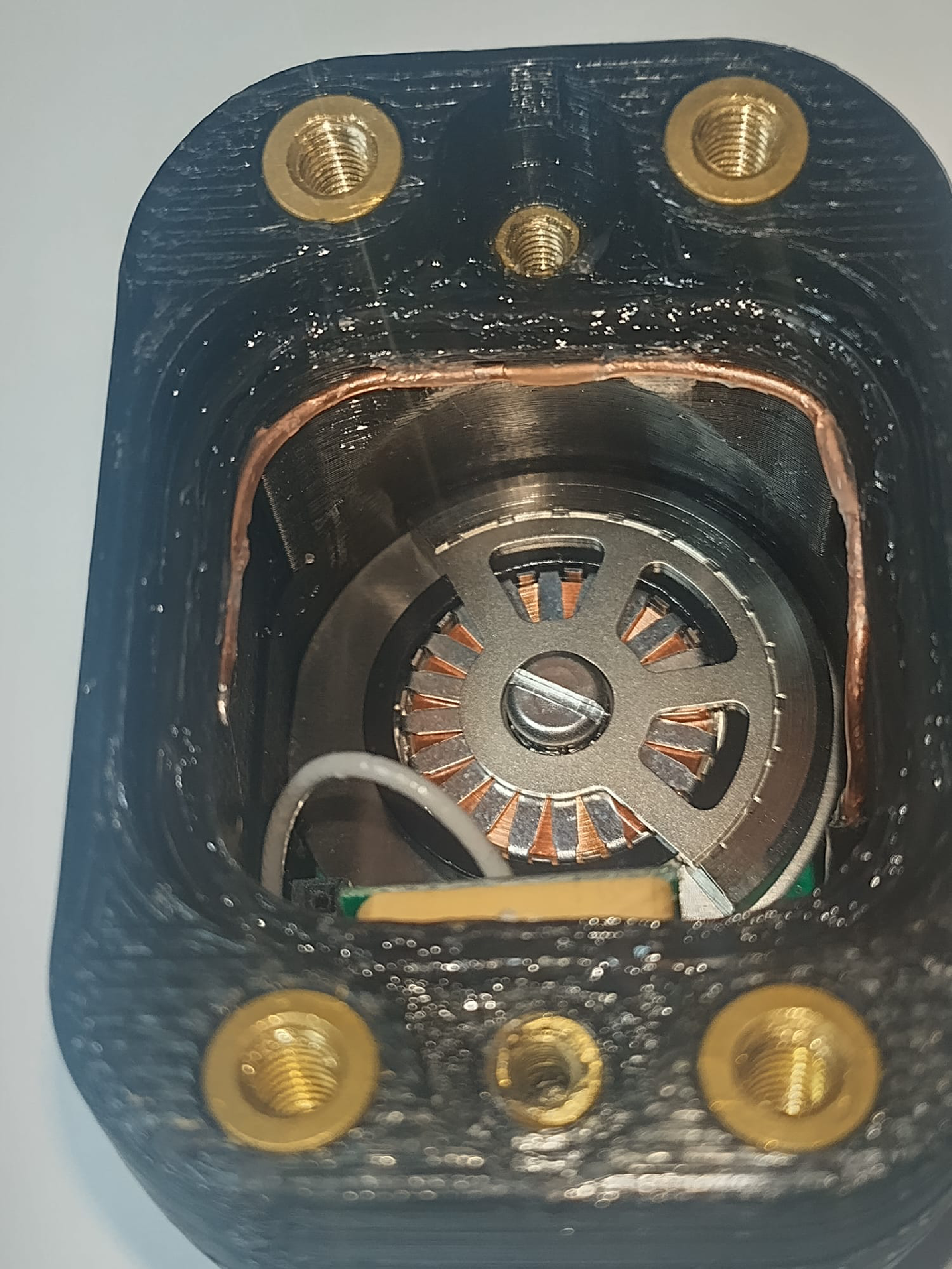

Supplement: S1 Fig — This is the version of the Kinefox V2 used in experiment 8 on the Exmoor pony. For the other experiments conducted with the Kinefox V2, the version showed in Fig 3 were used. (TIF) [file pone.0285930.s001.tif]

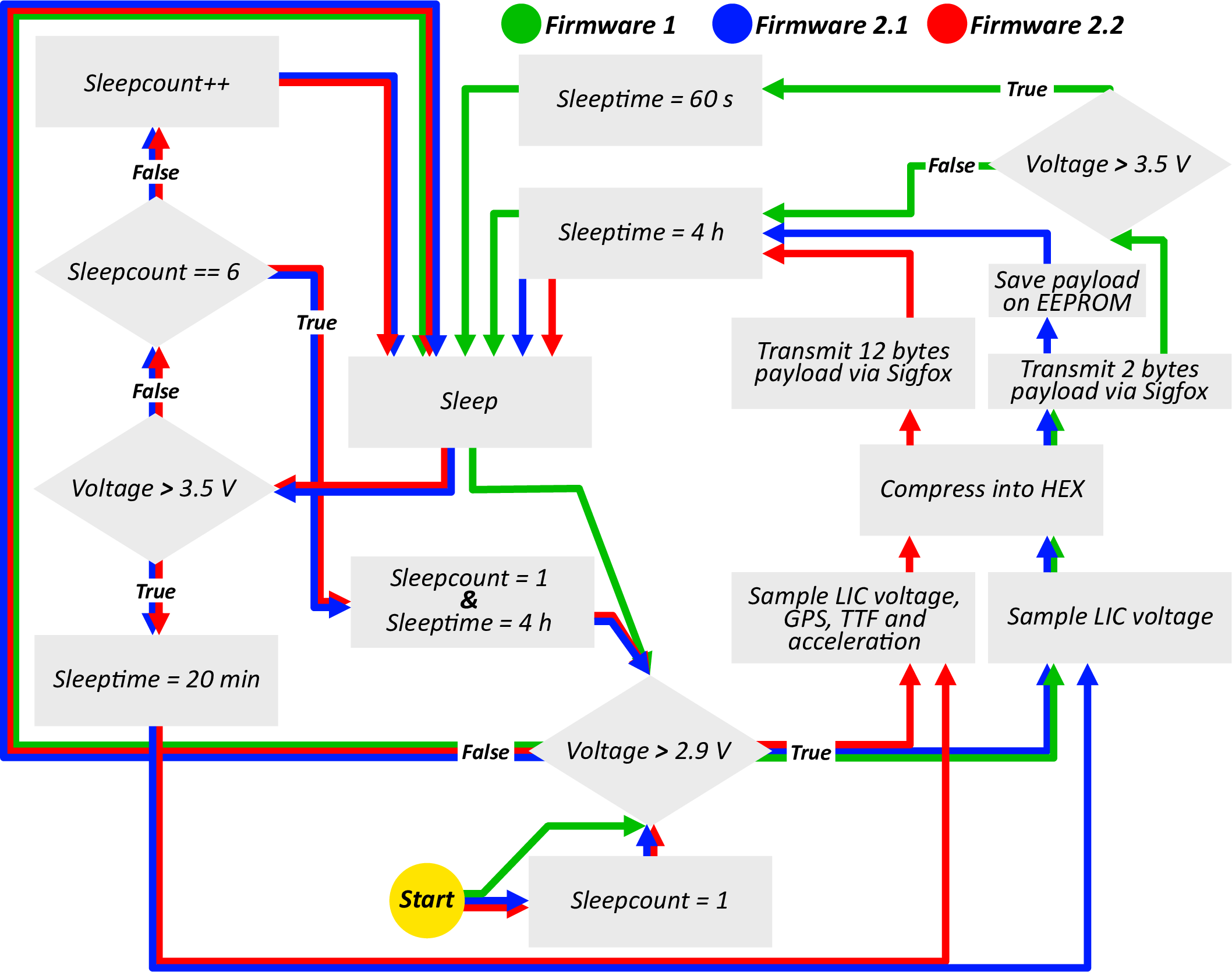

Supplement: S2 Fig — Flowchart illustrating the different firmware versions. Diamond shapes represent conditional statements, while the squares represent functions. (TIF) [file pone.0285930.s002.tif]

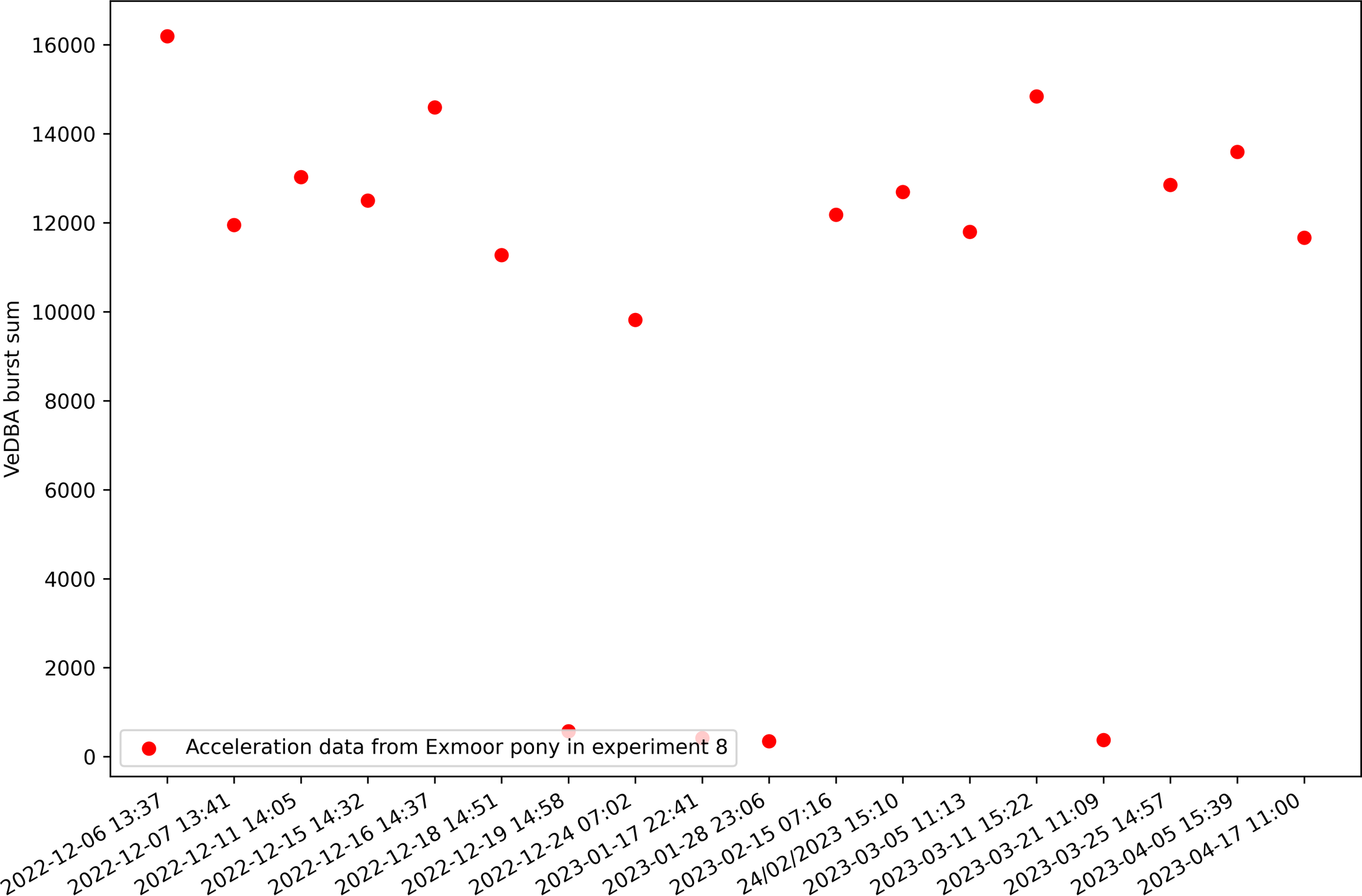

Supplement: S3 Fig — Data consists of the average VeDBA sum value sampled over 4 seconds at 54 Hz. The chart shows different levels of activity from low (VeDBA = 349) to high (VeDBA = 16191) probably indicating sleep, as sampling occurred during the dark hours of the night, and high activity in the form of walk/running/feeding respectably. This data confirms that the animal is alive and active. (TIF) [file pone.0285930.s003.tif]
