# Supplementary material for: A novel kinetic energy harvesting system for lifetime deployments of wildlife trackers
Source: PLoS One. 2023 May 17;18(5):e0285930. doi: 10.1371/journal.pone.0285930 (PMC10191315; doi:10.1371/journal.pone.0285930)
Supplement: S1 File — (DOCX) [file pone.0285930.s004.docx]

Contact information on the owner of the animals that the Kinefox prototypes were tested on.

All agreements were verbal and you are welcome to contact all the persons if you should have any questions.

Dog 1 (Balto): owner Peter Rask Møller (co-author), phone +45 20116643 email: [pdrmoller@snm.ku.dk](mailto:pdrmoller@snm.ku.dk)

Dog 2 and 4 (Pekka): owner Timm Wild (co-author), phone + 49 177 8454677 email: [twild@ab.mpg.de](mailto:twild@ab.mpg.de)

Wisent: owner Aage V. Jensens Foundation, Jacob Palsbøl Andersen, phone +45 20987382 email: [jpa@avjf.dk](mailto:jpa@avjf.dk)

Dog 3 (Comet): owner Shauhin Alavi, phone + 1 (760) 473 8781, email: [shauhinalavi@gmail.com](mailto:shauhinalavi@gmail.com)

Exmoor pony: owner Danish Nature Agency, Marianne Damholdt Bergin, +45 61797703, [mardb@nst.dk](mailto:mardb@nst.dk)
