# Supplementary material for: A novel kinetic energy harvesting system for lifetime deployments of wildlife trackers
Source: PLoS One. 2023 May 17;18(5):e0285930. doi: 10.1371/journal.pone.0285930 (PMC10191315; doi:10.1371/journal.pone.0285930)
Supplement: Attachment — Submitted filename: Review Comments to the Author.docx [file pone.0285930.s006.docx]

Review Comments to the Author

In this paper, the authors validate the use of a kinetic energy harvester as a power source for a wildlife tracker. The tracker is equipped with some sensors and a GPS and sends the data via Sigfox. In this work, it is tested on domestic dogs, a pony, and a wisent.

Overall, I find the paper original and I read it with interest. An aspect that should be adjusted is the structure of the paper and the proper allocation of the information in the chapters. A detailed description of the tests, equipment a methodology used, is, for example, missing in the method, a thing that makes the reading complicated.

Introduction, abstract, and discussion should be harmonized. Arguments that explain the unicity of the solution and the reasons why the proposed technology outperforms the existing ones should be addressed more clearly.

Finally, some imprecisions and not clear aspects are present in the results. Below you can find a detailed list of comments.

1. In the introduction, the authors analyze the advantages and disadvantages of alternative energy harvesters to support the choice of the kinetic harvester. They address the robustness issues of solar cells, with which I could agree, however, the robustness of the kinetic harvester is not addressed. Has the Kinetic harvester robustness issues, especially considering that it has mechanically moving parts and that must work outdoors with any temperature and weather?
2. Rows 85-88. Here the authors state that another problem of the solar cell is the reduction of efficiency over time, which makes them not suitable for several years of deployment. This statement is however later contradicted in raw 120-122. I suggest harmonizing the content. Also, I suggest that if performance over years is taken as a drawback for a specific technology, data on this aspect should be offered for the technology suggested in this paper. Is there available information on the aging of the MSG32?
3. Raw 160- 165. How does the max number of charge/discharge cycles of the supercap contribute to the presented device's lifetime?
4. In raw 113-119 the system components are only mentioned, without giving the components' names. Additionally, this part looks out of context in this part. I would suggest moving the text to the chapter “electronics”.
5. In the text, the targeted operation time should be stated clearly and without contradiction. Is the goal a lifetime tracking of the device (raw 36 and 104) or decades (raw 171)?
6. The authors should describe more clearly the differences between prototype V1 and V2 in the chapter electronics. In this chapter, it is clear the additional components in V2 with respect to V1. However, it is not clear which components are integrated into V1. The reader must get to the chapter embedded software before understanding that in v1 only the voltage of the LIC is sent.
7. The authors should integrate a scale in figure 3 so that the reader better understands how compact the device is.
8. The chapter case-study appears to be sound, however, the structure is unclear, making it difficult to follow. I advise the authors to rewrite this part to improve the readability of the text. A table listing the experiments, the prototype used, the period and the animal is helpful to improve the readability
9. The explanation of AVC, ANS, and AGP belongs to the methods, not in the description of table 1. The measurements tests and the parameters measured could be placed in “case-studies”. Additionally, it should be explained how the slope of figure 4 is the voltage change per day if the x-axes of figure 4 are not days but the number of transmissions. The variable AnimalAV is used in table 1 description for the first time without further explanation. To me, it is not clear the definition of this parameter as well as the formula for ANS. About the calculation of AGP, an explanation must be added. Are the authors able to know how many Sigfox messages are generated per day, or only the received messages are counted? Given that some messages get lost, by using the received messages, the energy consumption would be underestimated.
10. Raw 315-317. Could you please give here more details? Does the Kinefox still operate even if in sleep mode?
11. The measurement of energy consumption is shown and explained in the chapter “power consumption”. However, it is already used in the previous chapter (formulas in Tables 1 and 2). I suggest that the chapter “power consumption” is placed before the first use of measured values.
12. Part 328-334. Could the authors define what information was sent in the 2Bytes message, as they did for the 12-bytes message? Useful for the reader would be here to have a schematic representation of the payload.
13. For the power consumption experiments, the tests (instrumentations, protocol, etc.) must be described in the chapter Method.
14. In the discussion, it is addressed the difference between the collar materials used in prototype V1 and V2. This aspect must be addressed more clearly in the methods. As said in point 6 of this list a better explanation of the two prototypes is necessary for better comprehension.
15. Part 365- 383. The authors should explain in a more scientific way how and when the MSG32 outperforms batteries. The authors for example assert the necessity of their solution if 20J are used per day. Can the Authors explain, why exactly this value (which by the way was also not achieved in their work)? The Authors should make an energy comparison considering the features and energy consumption of devices on the market or from the literature.
16. The assertion in 381-383 is not supported by literature
17. The references should be checked. For some articles, the list of authors is not complete.
18. The text contains spelling errors (for example raw 73, 240, 241)
